# Characterization of Rift Valley Fever Virus MP-12 Strain Encoding NSs of Punta Toro Virus or Sandfly Fever Sicilian Virus

**DOI:** 10.1371/journal.pntd.0002181

**Published:** 2013-04-18

**Authors:** Olga A. Lihoradova, Sabarish V. Indran, Birte Kalveram, Nandadeva Lokugamage, Jennifer A. Head, Bin Gong, Bersabeh Tigabu, Terry L. Juelich, Alexander N. Freiberg, Tetsuro Ikegami

**Affiliations:** 1 Department of Pathology, The University of Texas Medical Branch, Galveston, Texas, United States of America; 2 Department of Microbiology and Immunology, The University of Texas Medical Branch, Galveston, Texas, United States of America; 3 Galveston National Laboratory, The University of Texas Medical Branch, Galveston, Texas, United States of America; 4 Sealy Center for Vaccine Development, The University of Texas Medical Branch, Galveston, Texas, United States of America; 5 Center for Biodefense and Emerging Infectious Diseases, The University of Texas Medical Branch, Galveston, Texas, United States of America; Tulane School of Public Health and Tropical Medicine, United States of America

## Abstract

Rift Valley fever virus (RVFV; genus *Phlebovirus*, family *Bunyaviridae*) is a mosquito-borne zoonotic pathogen which can cause hemorrhagic fever, neurological disorders or blindness in humans, and a high rate of abortion in ruminants. MP-12 strain, a live-attenuated candidate vaccine, is attenuated in the M- and L-segments, but the S-segment retains the virulent phenotype. MP-12 was manufactured as an Investigational New Drug vaccine by using MRC-5 cells and encodes a functional NSs gene, the major virulence factor of RVFV which 1) induces a shutoff of the host transcription, 2) inhibits interferon (IFN)-β promoter activation, and 3) promotes the degradation of dsRNA-dependent protein kinase (PKR). MP-12 lacks a marker for differentiation of infected from vaccinated animals (DIVA). Although MP-12 lacking NSs works for DIVA, it does not replicate efficiently in type-I IFN-competent MRC-5 cells, while the use of type-I IFN-incompetent cells may negatively affect its genetic stability. To generate modified MP-12 vaccine candidates encoding a DIVA marker, while still replicating efficiently in MRC-5 cells, we generated recombinant MP-12 encoding Punta Toro virus Adames strain NSs (rMP12-PTNSs) or Sandfly fever Sicilian virus NSs (rMP12-SFSNSs) in place of MP-12 NSs. We have demonstrated that those recombinant MP-12 viruses inhibit IFN-β mRNA synthesis, yet do not promote the degradation of PKR. The rMP12-PTNSs, but not rMP12-SFSNSs, replicated more efficiently than recombinant MP-12 lacking NSs in MRC-5 cells. Mice vaccinated with rMP12-PTNSs or rMP12-SFSNSs induced neutralizing antibodies at a level equivalent to those vaccinated with MP-12, and were efficiently protected from wild-type RVFV challenge. The rMP12-PTNSs and rMP12-SFSNSs did not induce antibodies cross-reactive to anti-RVFV NSs antibody and are therefore applicable to DIVA. Thus, rMP12-PTNSs is highly efficacious, replicates efficiently in MRC-5 cells, and encodes a DIVA marker, all of which are important for vaccine development for Rift Valley fever.

## Author Summary

Upon outbreak of zoonotic viral diseases in herds of animals, early detection of naturally infected animals and prevention of further viral spread are important for minimizing the impact of outbreak in the society. Vaccination may compromise the identification of infected animals since both natural infection and vaccination induce antibodies specific to the pathogen. Therefore, new generation vaccines should have a marker to differentiate infected from vaccinated animals (DIVA). Rift Valley fever virus (RVFV) is a mosquito-borne zoonotic pathogen which can cause hemorrhagic fever, neurological disorders or blindness in humans and a high-rate abortion in ruminants. MP-12 strain, a live-attenuated candidate vaccine, is safe and immunogenic, but lacks a DIVA marker. In this study, we developed and characterized improved MP-12 viruses which encode a DIVA marker by replacing the virulence gene with that of serologically distinct viruses belonging to the same genera. The novel MP-12 variant with such DIVA marker was highly efficacious and replicated efficiently in human diploid cells for vaccine production, and will become alternative candidate vaccines of MP-12 for veterinary applications.

## Introduction

Rift Valley fever virus (RVFV), which belongs to the family *Bunyaviridae*, genus *Phlebovirus*, is a zoonotic pathogen transmitted by mosquitoes, and the causative agent for Rift Valley fever (RVF). RVF is characterized by a high rate of abortion and fetal malformation in pregnant ruminants, febrile illness in adult ruminants, and lethal acute hepatitis in newborn lambs [1]. In humans, patients suffer an acute febrile illness with occasional complications including partial or complete blindness, hemorrhagic fever, or neurological disorders [2], [3], [4], [5]. RVFV can be transmitted through the drought-resistant eggs of infected floodwater *Aedes* mosquitoes which thus play a role in maintaining RVFV in endemic areas. Other mosquito species are also involved in RVFV transmission if RVFV-infected mosquito population increases subsequent to heavy rain fall or increase in mosquito habitats [6], [7], [8], [9]. RVF has been endemic to sub-Saharan Africa, and has spread into Madagascar, Comoro, Egypt, Saudi Arabia and Yemen [10], [11], [12], [13], [14], [15], [16], [17]. The development of an effective vaccine against RVF is important for non-endemic countries to prevent further spread of RVFV. RVFV is classified as an NIAID Category A Priority Pathogen and an overlap select agent by the U.S. Department of Health and Human Services (HHS) and Agriculture (USDA) [18]. RVFV is transmitted via aerosol, and the handling of virus should be done in biosafety level (BSL) 3+ or 4 laboratories.

RVFV has a tripartite negative-stranded RNA genome composed of Small (S)-, Medium (M)- and Large (L)-segments. The S-segment encodes N and NSs genes in an ambi-sense manner, the M-segment contains a single open reading frame (ORF) which encodes NSm, 78-kD protein, NSm-Gn, Gn, and Gc proteins from different AUGs and co-translational cleavage; and the L-segment encodes RNA-dependent RNA polymerase [19], [20], [21], [22]. The nonstructural protein, NSs is a major virulence factor, and it inhibits host general transcription by inhibiting host basal transcription factor (TF) IIH; TFIIH is one of six general transcription factors (TFIIA, TFIIB, TFIID, TFIIE, TFIIF and TFIIH) [23], composed of 10 different proteins; i.e., XPD, XPB, p8, p34, p44, p52, p62, MAT1, cyclin H and cdk7 [24], [25], and essential for RNA synthesis by cellular RNA polymerase I and II [26], [27]. NSs binds to and sequester TFIIH p44 [28] and also promotes the degradation of TFIIH p62 [29]. On the other hand, NSs inhibits the activation of interferon (IFN)-β promoter by interacting with Sin3A-associated protein (SAP30) and recruiting repressor complex containing histone deacetylase-3 (HDAC-3) [30], [31], while NSs promotes the degradation of dsRNA-dependent protein kinase, PKR [32], [33], [34].

Smithburn vaccine generated by mouse brain passages of RVFV Entebbe strain has been sold as a veterinary vaccine in South Africa, Kenya, Zimbabwe, Namibia, Egypt and Israel [11]. This vaccine itself caused abortion in pregnant ruminants and also reassorted with natural wild-type (wt) RVFV due to the use during an outbreaks [11]. MP-12 vaccine was generated by 12 serial plaque-passages in human diploid MRC-5 cells in the presence of the chemical mutagen 5-fluorouracil [35], [36]. MP-12 is highly immunogenic in ruminants, and can also induce sufficient immune response in humans [37], [38], [39], [40], [41]. MP-12 is excluded from the select agent rule in the U.S., and can be handled in BSL-2 laboratories. MP-12 vaccine was manufactured by using certified MRC-5 cells as an Investigational New Drug for human clinical trials [36]. It is known that RVFV causes spontaneous truncation of NSs gene during passages in mammalian Vero or BHK-21 cells which lack functional type-I IFN system [42] or in *Aedes aegypti* larvae (Aag2) cells [43], [44]. We recently characterized the genetic subpopulations of MP-12 vaccine Lot 7-2-88 and found that MP-12 vaccine retains highly stable attenuation mutations in the M- and L-segments during its cultivation in MRC-5 cells [36]. Since MP-12 is attenuated by only point mutations at M- and L-segments, a potential of reversion to virulence cannot be excluded, and MP-12 requires further improvement for veterinary use. Another concern is a lack of DIVA (Differentiating Infected from Vaccinated Animals) markers. In previous study, we found 27% of mice vaccinated with MP-12 induce detectable anti-NSs antibody [45]. Though the immunogenicity of MP-12 NSs is poor, the presence of anti-NSs antibody in vaccinated group will compromise DIVA strategy. Without DIVA markers, it is impossible to monitor infected animals in herds of vaccinated ruminants during RVF outbreaks.

In this study, we aimed to develop MP-12 variants which encode a DIVA marker and replicate efficiently in MRC-5 cells. Although recombinant MP-12 lacking NSs gene in the S-segment encodes a negative DIVA marker [45]; i.e., a lack of anti-NSs antibody response, it does not replicate efficiently in type-I IFN-competent MRC-5 cells. The *Phlebovirus* genus consists of the sandfly fever group including serologically distinct Punta Toro serocomplex, Naples serocomplex, Icoaraci serocomplex, Frijoles serocomplex, Sicilian serocomplex, RVFV, and the Uukuniemi group [46], and some of the different phlebovirus NSs are reported to be able to interfere with the host type-I IFN system [34], [47], [48]. In this study, we developed recombinant MP-12 encoding NSs of Punta Toro virus Adames strain (PTV) (rMP12-PTNSs) or Sandfly fever Sicilian virus (SFSV) (rMP12-SFSNSs) in place of MP-12 NSs [46]. It should be noted that a lack of MP-12 NSs serves as a negative DIVA marker to identify animals exposed to wt RVFV, and we did not attempt to identify vaccinated animals by detecting antibody specific to anti-PTV NSs or anti-SFSV NSs. We characterized the functions of those NSs, and determined the immunogenicity and efficacy of rMP12-PTNSs and rMP12-SFSNSs in the outbred CD1 mouse model. Our results suggested that rMP12-PTNSs, but not rMP12-SFSNSs, replicates efficiently in MRC-5 cells, while both are as efficacious as parental MP-12, and also did not induce antibodies cross-reactive to RVFV NSs. Thus, rMP12-PTNSs is an alternative candidate vaccine which can be amplified in MRC-5 cells and encodes a negative DIVA marker.

## Materials and Methods

### Media, cells and viruses

VeroE6 cells (ATCC CRL-1586), 293 cells (ATCC CRL-1573), MRC-5 cells (ATCC CCL-171) and MEF cells [49] were maintained in Dulbecco's modified minimum essential medium (DMEM) containing 10% fetal calf serum (FCS). BHK/T7-9 cells that stably express T7 RNA polymerase [50] were maintained in MEM-alpha containing 10% FCS with 600 µg/ml of hygromycin. Penicillin (100 U/ml) and streptomycin (100 µg/ml) were added to the culture media. MP-12 vaccine Lot 7-2-88 (kindly provided from Dr. J.C. Morrill at the University of Texas Medical Branch: UTMB) was amplified twice in MRC-5 cells for experiments. rMP12-PTNSs, rMP12-PTNSs-Flag, rMP12-SFSNSs and rMP12-SFSNSs-Flag were rescued from plasmid DNAs in BHK/T7-9 cells as described previously [51], and passaged once in VeroE6 cells. rMP12-NSsR173A and rMP12-NSs-Flag were reported previously [32], [52]. RVFV ZH501 strain stock was generated after one VeroE6 cell passage of an original ZH501 reference collection vial (Serial #JM1137) at UTMB [53]. Sendai virus Cantell strain was purchased from Charles River (North Franklin, CT).

### Plasmids

The plasmid encoding anti-viral-sense of MP-12 S-segment at the downstream of the T7 promoter, pProT7-S(+), was described previously [51]. VeroE6 cells were infected with PTV Adames strain or SFSV Sabin strain (provided by Dr. R.B. Tesh, UTMB), and the total RNA was extracted at 3 dpi. First stranded cDNA was synthesized with Superscript II Reverse Transcriptase (Invitrogen), and the NSs ORF was amplified with Phusion DNA polymerase (New England Biolabs) by using specific primers with uniquely incorporated *Hpa*I and *Spe*I restriction sites for cloning. The PCR fragments of PTV Adames or SFSV NSs were cloned into pProT7-S(+) [51] in place of MP-12 NSs, designated as pProT7-S(+)PTNSs or pProT7-S(+)SFSNSs, respectively. Similarly, Flag-tag was added at the C-terminus of those NSs, and the resulting plasmids were designated as pProT7-S(+)PTNSs-Flag or pProT7-S(+)SFSNSs-Flag, respectively. The pcDNA3.1mycHisA plasmids encoding CAT [32] or NSs of PTV Adames or SFSV NSs (without tag) were generated, and designated as pcDNA3.1mycHisA-PTNSs or pcDNA3.1mycHisA-SFSVNSs. For reporter assay, IFNb-pGL3 plasmid was kindly provided by Dr. R. Lin at McGill Univ. [54], 4×IRF3-luc plasmid was kindly provided by Dr. S. Ludwig at ZMBE, Westfälische-Wilhelms-University [55], and pPRDII-luc plasmid was kindly provided by Dr. M. Gale Jr. at Univ. of Washington [56]. The pRL-SV40 plasmid was purchased from Promega (Madison, WI).

### Recovery of recombinant MP-12

The recombinant MP-12 encoding NSs truncation or mutation were recovered by using a plasmid combination of pProT7-M(+), pProT7-L(+), pT7-IRES-vN, pT7-IRES-vL, pCAGGS-vG and either of pProT7-S(+)PTNSs or pProT7-S(+)SFSNSs. BHK/T7-9 cells were transfected with those plasmids as described previously [51]. Recombinant MP-12 encoding NSs of PTV Adames or SFSV were designated as rMP12-PTNSs or rMP12-SFSNSs, respectively. Recovered recombinant MP-12 were amplified once in VeroE6 cells, titrated by plaque assay, and used for experiments. In addition, we also generated recombinant MP-12 encoding C-terminus Flag-tagged NSs of PTV Adames or SFSV, and those mutants were designated as rMP12-PTNSs-Flag or rMP12-SFSNSs-Flag, respectively.

### Northern blot analysis

Total RNA was extracted from infected or mock-infected cells using TRIzol reagent. Denatured RNA was separated on 1% denaturing agarose-formaldehyde gels and transferred onto a nylon membrane (Roche Applied Science, Indianapolis, IN). Northern blot analysis was performed as described previously with strand-specific RNA probes to detect RVFV anti-sense S-segment/N mRNA, mouse IFN-β mRNA, or mouse ISG56 mRNA [57].

### Western blot analysis

Western blot analysis was performed as described previously [32]. The membranes were incubated with anti-human PKR monoclonal antibody (BD Biosciences), anti-mouse PKR monoclonal antibody (B-10, Santa Cruz, CA), anti-RVFV mouse polyclonal antibody (a kind gift from Dr. R.B.Tesh, UTMB), anti-Flag-tag M2 monoclonal antibody (Sigma), or anti-β-actin goat polyclonal antibody (I-19; Santa Cruz, CA.) overnight at 4°C and with secondary antibodies (Santa Cruz, CA) for 1 hr at room temperature.

### Analysis of virus replication

MRC-5 cells were infected with MP-12, rMP12-C13type, rMP12-PTNSs or rMP12-SFSNSs at a m.o.i of 0.01 at 37°C for 1 h, washed cells twice with media, and incubated at 37°C. Culture supernatants were collected at 0 (after removal of viral inocula), 24, 48, 72 and 96 hpi, and used for plaque assay [51], [58]. Viral titers in culture supernatants from VeroE6 cells or MEF cells infected with those viruses at a m.o.i of 0.01, which were collected at 72 hpi, were also titrated.

### In vitro RNA synthesis

The pcDNA3.1mycHisA plasmids encoding CAT [32] or pcDNA3.1mycHisA-PTNSs or pcDNA3.1mycHisA-SFSVNSs were linearized, and in vitro transcribed by using mMESSAGE mMACHINE T7 Ultra kit (Ambion, Grand Island, NY) according to the manufacturer's instructions. The linearized CAT DNA contained myc-His tag at the 3′end.

### Transfection

Transfection of plasmid DNA or *in vitro* synthesized RNA was performed by using TransIT-LT1 or TransIT-mRNA Transfection Kit (Mirus, Madison, WI) according to manufacturer's instructions, respectively.

### Reporter assay

293 cells in 12-well plate were transfected with 1 µg of IFNb-pGL3 plasmids, 4×IRF3-luc plasmid, or pPRDII-luc plasmids in addition to 0.1 µg of pRL-SV40 plasmid. At 24 hours post transfection, cells were mock-infected or infected with 300 µl of SeV (100 U/ml), and mock-transfected or immediately transfected with 1 µg of in vitro synthesized RNA encoding CAT (control) or NSs of MP-12, PTV Adames strain or SFSV. Cells were collected at 16 hpi, and the relative luciferase activity was measured by Dual-Luciferase Reporter Assay System (Promega, Madison, WI) according to manufacturer's instructions.

### Analysis of general transcription

The analysis of host general transcription suppression was described previously [29]. Briefly, 293 cells were mock-infected or infected with either MP-12, rMP12-PTNSs or rMP12-SFSNSs at a m.o.i of 3. Cells were treated with 0.5 mM 5-ethynyluridine (EU) from 12 to 13 hpi before harvesting at 13 hpi. As a control for transcriptional suppression, cells were treated with 5 µg/ml of ActD concurrently with the EU treatment. Incorporated EU was detected with an AlexaFluor 647-coupled azide (Invitrogen), and viral antigens were stained with anti-RVFV antibodies followed by an AlexaFluor 488-coupled secondary antibody. Cells were analyzed by flow cytometry on an LSRII Fortessa instrument (BD Biosciences).

### Immunization and virus challenge

For testing the efficacy of MP-12 NSs mutants, 5-week-old female CD1 outbred mice (Charles River, North Franklin, CT) were inoculated subcutaneously with PBS (mock) (n = 10) or 1×10^5^ pfu of MP-12 (n = 20), rMP12-NSR173A (n = 10), rMP12-PTNSs (n = 9) or rMP12-SFSNSs (n = 10). Those mice were challenged with 1×10^3^ pfu of wt RVFV ZH501 strain (i.p) at 45 days post vaccination. The challenge experiment was performed at an animal biosafety level 4 facility at the UTMB Shope laboratory. Mice were observed for 21 days after challenge, and the body weight was monitored daily. Sera were collected at 1, 2, 3, and 42 days post vaccination (retro-orbital bleeding), and at 21 days post wt RVFV challenge (cardiac puncture). Survival curves of mice (Kaplan-Meyer method) were analyzed by Graphpad Prism 5.03 program (Graphpad Software Inc, La Jolla, CA.).

### Plaque reduction neutralization test

The PRNT_80_ was determined as described previously [45]. Briefly, Each 20-µl of mouse sera serially diluted 4-fold was transferred into flat-bottom 96-well plates containing 5 µl of MP-12 virus (50 pfu/well) (final dilutions of sera: 1∶10, 1∶40, 1∶160∼). After incubation at 37°C for 1 hour, 150 µl of DMEM with 10% FBS was added to the well. The 150 µl of the mixture was transferred into a 24-well plate with confluent VeroE6 cells, and the plate was incubated at 37°C for 1 hour. After removal of inocula, virus titer was determined by plaque assay. Eighty % of the average number of plaques in 6 different wells that had mock-immunized mice sera was used as the cut-off number (typically 8∼9). The highest dilution of sera that produced the number of plaques below the cut-off number was designated as the PRNT_80_ neutralizing antibody titer.

### IgG-ELISA

His-tagged RVFV N proteins [45], which were expressed and purified using recombinant baculovirus, or purified GST-tagged RVFV NSs C-terminal 48 amino acids [45], which were expressed and purified using *E,coli*, were coated onto 96-well ELISA plates overnight at 4°C at a concentration of 100 ng/well. After washing 3 times with PBS containing 0.1% Tween 20 (PBS-T), the wells were blocked with PBS-T containing 5% skim milk at 37°C for 2 hours. Then, wells were incubated with serum samples (2-fold dilutions for anti-N IgG, and 1∶100 for anti-NSs IgG) at 37°C for 1 hour. Wells were washed for 3 times with PBS-T and reacted with HRP-conjugated anti-mouse IgG (Santa Cruz, CA) at 37°C for 1 hour. After washing with PBS-T for 3 times, ABTS was added to wells. The plate was incubated at room temperature for 30 min, and the optical density (OD) at 405 nm was recorded. The cut-off value, 0.176, was defined as mean+2×standard deviation of 24 normal mouse serum samples (1∶400) for anti-N IgG, while the cut-off value of 0.204 was defined as mean+3×standard deviation of 24 normal mouse serum samples. The highest dilution of sera that made a OD value larger than the cut-off was designated as the anti-N antibody titer. Because the anti-NSs antibody level was low, an OD value of a 1∶100 dilution was used for demonstrating the presence of anti-NSs antibody.

### Statistical analysis

Statistical analyses were performed by using the Graphpad Prism 5.03 program (Graphpad Software Inc, La Jolla, CA). Unpaired t-test or Mann-Whitney U-test was used for the comparison of two groups. Survival curves of mice were analyzed by log-rank (Mantel-Cox) test.

### Ethics statement

Mouse studies were performed in facilities accredited by the Association for Assessment and Accreditation of Laboratory Animal Care (AAALAC) in accordance to the Animal Welfare Act, NIH guidelines, and US federal law. The animal protocol was approved by the University of Texas Medical Branch (UTMB) Institutional Animal Care and Use Committee (IACUC) (protocol #1007038). All the recombinant DNA and RVFV were created upon the approval of the Notification of Use by the Institutional Biosafety Committee at UTMB. The wt RVFV ZH501 strain was used at the Robert E. Shope BSL4 laboratory at the UTMB in accordance with NIH guidelines and US federal law.

## Results

### Generation of recombinant MP-12 encoding NSs of PTV or SFSV

**Figure 1:**
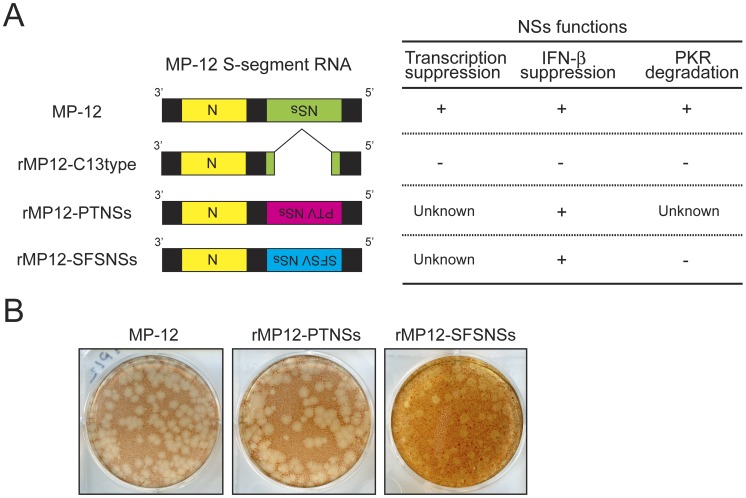
Generation of rMP12-PTNSs and rMP12-SFSNSs. (A) Schematics of MP-12 S-segments encoding mutation or foreign gene in place of MP-12 NSs. The rMP12-C13type (C13type) lacks 69% of the NSs ORF as described previously [44]. The rMP12-PTNSs, and rMP12-SFSNSs encode NSs of Punta Toro virus Adames strain and Sandfly fever Sicilian virus, respectively. The expected phenotype corresponding to each S-segment is also presented. (B) Plaque phenotypes of MP-12, rMP12-PTNSs and rMP12-SFSNSs at 4 dpi. Plaque assay was performed with VeroE6 cells overlaid with 0.6% noble agar and stained with Neutral red.

In this study, we aimed to develop a modified MP-12 vaccine encoding NSs derived from serologically distinct phleboviruses. We attempted to select the phlebovirus NSs which can inhibit type-I IFN induction. Using reverse genetics for RVFV MP-12 strain, we recovered recombinant MP-12 encoding NSs of PTV Adames strain (rMP12-PTNSs) or SFSV (rMP12-SFSNSs) (**Fig. 1A**). In VeroE6 cells, rMP12-PTNSs formed clear plaques similar to those of MP-12, while rMP12-SFSNSs formed turbid plaques similar to those of rMP12-C13type [51], [58] (**Fig. 1B**). PTV Adames strain NSs [48] and SFSV NSs inhibit IFN-β gene [34], while SFSV NSs does not promote the degradation of PKR [34].

**Figure 2:**
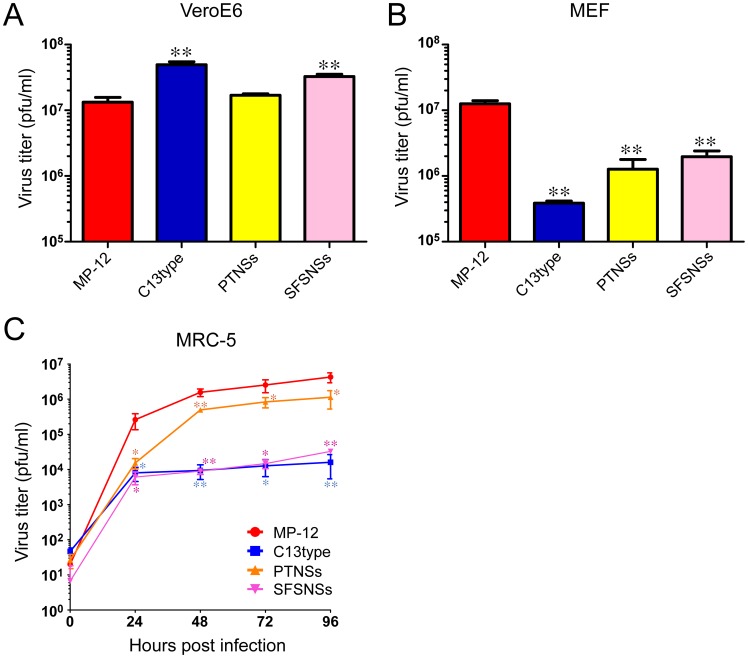
Replication of rMP12-PTNSs and rMP12-SFSNSs in cell culture. (A) VeroE6 cells, (B) MEF cells or (C) MRC-5 cells were mock-infected or infected with MP-12, rMP12-C13type, rMP12-PTNSs, or rMP12-SFSNSs at a m.o.i of 0.01. Culture supernatants were collected at 72 hpi (A and B), or indicated time points (C) and virus titer was determined by plaque assay with VeroE6 cells. Means+standard deviations of three independent experiments are shown in the graph. Asterisk represents statistical significance (Unpaired t-test, **p<0.01, vs. MP-12).

We first tested the replication capability of those viruses in type-I IFN incompetent VeroE6 cells and mouse embryonic fibroblast (MEF) cells (**Fig. 2A and B**). Cells were infected with the indicated virus at a multiplicity of infection (m.o.i) of 0.01, and culture supernatants were collected at 72 hpi for viral titration. In VeroE6 cells, MP-12 and rMP12-PTNSs replicated to a similar level, while rMP12-C13type and rMP12-SFSNSs replicated slightly more efficiently than MP-12 (**Fig. 2A**). In MEF cells at 72 hpi, MP-12 replicated 1.51, 0.99 or 0.81 log more than rMP12-C13type, rMP12-PTNSs or rMP12-SFSNSs, respectively (**Fig. 2B**). Subsequently, we determined viral growth kinetics in MRC-5 cells, because MRC-5 cells were used for the cultivation of MP-12 vaccine [36]. In MRC-5 cells, rMP12-SFSNSs did not replicate efficiently, and the replication kinetics was similar to that of rMP12-C13type, while rMP12-PTNSs replicated efficiently at the level nearly similar to that of MP-12 (**Fig. 2C**). At 72 hpi, MP-12 replicated 2.27, 0.54 or 2.14 log more than rMP12-C13type, rMP12-PTNSs or rMP12-SFSNSs, respectively (**Fig. 2C**).

### PKR degradation by MP-12 encoding NSs of PTV or SFSV

**Figure 3:**
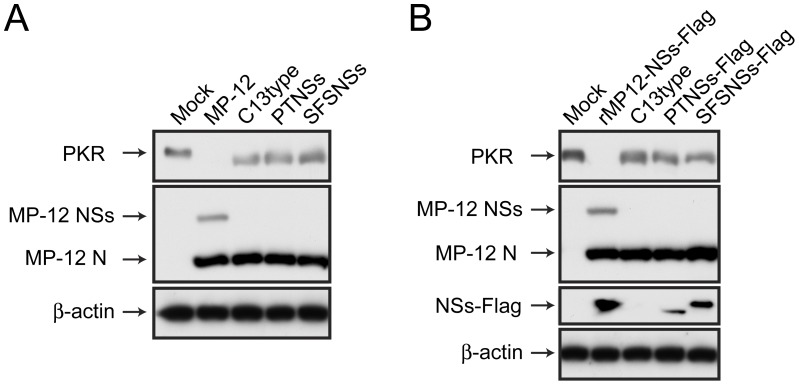
Degradation of PKR in VeroE6 cells infected with rMP12-PTNSs or rMP12-SFSNSs. (A) VeroE6 cells were mock-infected or infected with MP-12, rMP12-C13type, rMP12-PTNSs or rMP12-SFSNSs at a m.o.i of 3, and cells were collected at 16 hpi. Western blot was performed with anti-PKR, anti-RVFV and anti-β-actin antibodies. (B) VeroE6 cells were mock-infected or infected with rMP12-NSs-Flag [32], rMP12-C13type, rMP12-PTNSs-Flag or rMP12-SFSNSs-Flag at a m.o.i of 3, and Western blot was performed as described above. Anti-Flag antibody was used for the detection of NSs-Flag. Representative data from three independent experiments are shown.

RVFV NSs promotes the degradation of PKR thus inhibiting the phosphorylation of eIF2α, which promotes viral protein synthesis [32], [52]. To understand the functional difference between RVFV NSs and other phlebovirus NSs, we first tested whether they degraded PKR. To avoid the up-regulation of PKR by type-I IFN, we used type-I IFN-incompetent VeroE6 cells [59], [60]. VeroE6 cells were mock-infected or infected with MP-12, rMP12-C13type, rMP12-PTNSs or rMP12-SFSNSs at a m.o.i of 3 and cells were collected at 16 hpi for Western blot analysis (**Fig. 3A**). As expected, cells infected with MP-12 promoted the degradation of PKR, while cells infected with MP-12 encoding NSs of PTV or SFSV expressed abundant PKR at 16 hpi. The NSs accumulation of rMP12-PTNSs or rMP12-SFSNSs could not be detected by mouse polyclonal antibodies against PTV or SFSV probably due to the sensitivity of the antibody to detect NSs (data not shown). To evaluate the level of each NSs accumulation, NSs of PTV or SFSV were fused to Flag-tag at the C-terminus, and the resulting viruses were designated as rMP12-PTNSs-Flag or rMP12-SFSNSs-Flag, respectively. As a control for NSs-Flag expression, we also used rMP12-NSs-Flag, which encodes an MP-12 NSs with a C-terminus Flag-tag [32]. VeroE6 cells were mock-infected or infected with rMP12-NSs-Flag, rMP12-C13type, rMP12-PTNSs-Flag or rMP12-SFSNSs-Flag at a m.o.i of 3, collected at 16 hpi, and subjected to Western blot analysis using anti-Flag antibody. As shown in **Fig. 3B**, we confirmed that NSs of PTV Adames and SFSV were expressed at 16 hpi. Collectively, we concluded that the rMP12-PTNSs and rMP12-SFSNSs do not promote the degradation of PKR.

### General transcription suppression by MP-12 encoding NSs of PTV or SFSV

**Figure 4:**
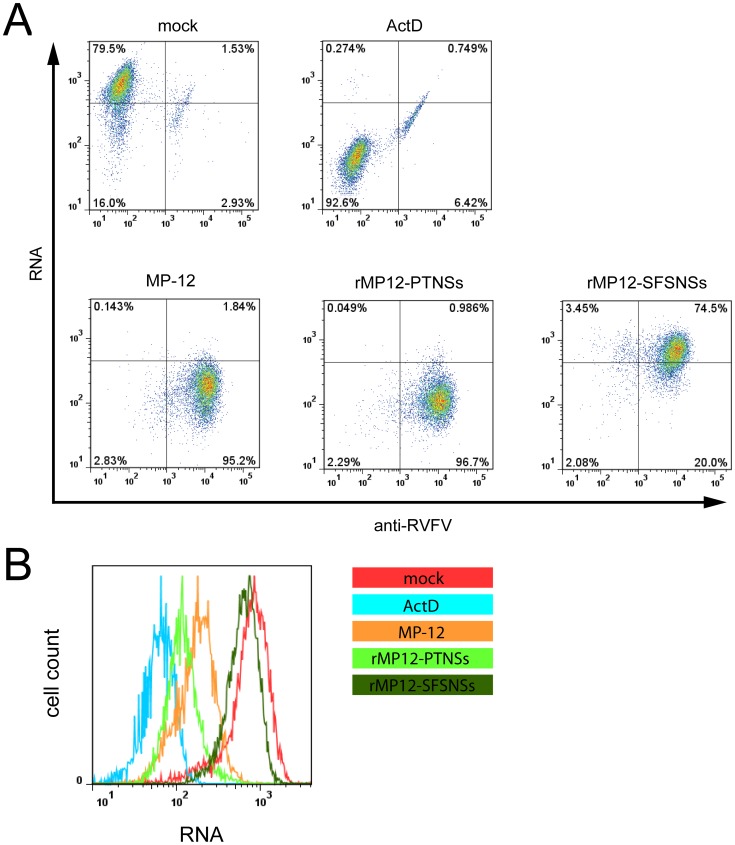
Host general transcriptional suppression by RVFV MP-12 mutants. (A) Flow cytometry analysis was performed in 293 cells. 293 cells were mock-infected or infected with MP-12, rMP12-C13type, rMP12-PTNSs or rMP12-SFSNSs at a m.o.i of 3 and treated with 0.5 mM EU at 8 hpi for 3 hours. Mock-infected cells were co-treated with ActD (5 µg/ml) at 8 hpi for 3 hours. Incorporated EU was stained with Alexa Fluor 647-azide, and RVFV antigens were stained with anti-RVFV antibodies and detected by Alexa Fluor 488 anti-mouse IgG. Subsequently, cells were analyzed by flow cytometry. Representative data from two independent experiments are shown. X-axis: signal intensity for RVFV antigen, Y-axis: signal intensity for EU. (B) Relative fluorescence intensity of EU-positive cells is shown as a histogram.

We found that both the rMP12-PTNSs and rMP12-SFSNSs do not promote the degradation of PKR. To clarify whether the rMP12-PTNSs or rMP12-SFSNSs can induce host general transcription suppression, 293 cells were mock-infected or infected with MP-12, rMP12-PTNSs or rMP12-SFSNSs at a m.o.i of 3, and nascent RNA was labeled with 5-ethynyluridine (EU) [61], a uridine analog, from 12 to 13 hpi. As a control for transcriptional suppression, mock-infected cells were incubated with actinomycin D (ActD) (5 µg/ml) concurrently with the EU treatment. The incorporated EU was covalently linked to azide conjugated with AlexaFluor 647, and cells were further stained with anti-RVFV antibody to detect expression of viral proteins [29]. Then, the level of EU-incorporation and expression of viral proteins were analyzed by flow cytometry. **Fig. 4A** depicts the acquired data as a dot plot with the expression of viral proteins (anti-RVFV) on the x-axis and the incorporation of EU (RNA) on the y-axis. Quadrant gates were set to that the majority of mock infected cells (79.5%) were in the upper left quadrant, the majority of ActD treated cells (92.6%) in the lower left quadrant and the majority of anti-RVFV positive cells in either the right upper or lower quadrant. When cells were infected with MP-12, 95.2% of total cells (or 98.1% of anti-RVFV positive cells) showed reduced EU incorporation when compared to mock infected cells. Similarly, 98.99% of anti-RVFV positive cells showed reduced EU incorporation when cells were infected with rMP12-PTNSs. In contrast, when cells were infected with rMP12-SFSNSs, 78.84% of anti-RVFV positive cells still incorporated EU at the same level as mock infected cells. **Fig. 4B** depicts the level of EU incorporation of the anti-RVFV negative population (mock and ActD) and anti-RVFV positive population (MP-12, rMP12-PTNSs and rMP12-SFSNSs infected cells) as a histogram where the RNA fluorescence intensity (EU incorporation) is plotted on the x-axis and the cell count is plotted on the y-axis. These data suggest that MP-12 and rMP12-PTNSs are able to suppress host transcription, whereas rMP12-SFSNSs has no negative effect on host RNA synthesis.

### IFN-β gene suppression by MP-12 encoding NSs of PTV or SFSV

**Figure 5:**
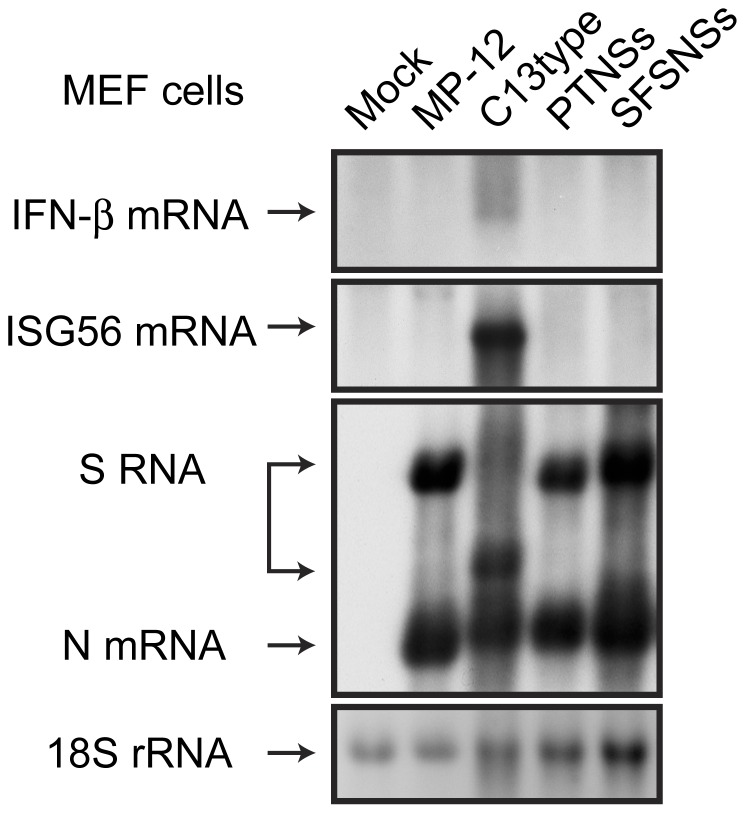
rMP12-PTNSs and rMP12-SFSNSs inhibit IFN-β mRNA synthesis. MEF cells were mock-infected or infected with MP-12, rMP12-C13type, rMP12-PTNSs or rMP12-SFSNSs at a m.o.i of 3. Total RNA was harvested at 7 hpi. Northern blotting was performed with strand-specific RNA probes to detect mouse IFN-β or ISG56 mRNA, or RVFV anti-sense S-segment/N mRNA, respectively. The 18S rRNA was shown as loading control. Representative data from three independent experiments are shown.

We found that rMP12-PTNSs but not rMP12-SFSNSs induces host general transcription suppression. To know whether rMP12-PTNSs or rMP12-SFSNSs can inhibit IFN-β gene up-regulation, type-I IFN-competent MEF cells were mock-infected or infected with MP-12, rMP12-C13type, rMP12-PTNSs or rMP12-SFSNSs at a m.o.i of 3, and total RNA was extracted at 7 hpi. Accumulation of mouse IFN-β, ISG56 mRNA, RVFV antiviral-sense S-segment RNA and N mRNA was analyzed by Northern blot as described previously [51], [62]. Cells infected with rMP12-C13type induced IFN-β and ISG56 mRNA, while those infected with MP-12, rMP12-PTNSs or rMP12-SFSNSs did not induce IFN-β and ISG56 mRNA synthesis (**Fig. 5**). These results suggest that both PTV and SFSV NSs inhibit the accumulation of IFN-β mRNA, consistent with previous studies [34], [48]. Taken together, the results suggest that rMP12-PTNSs inhibits both host general transcription and IFN-β mRNA synthesis and that rMP12-SFSNSs inhibits IFN-β mRNA synthesis but not host general transcription.

**Figure 6:**
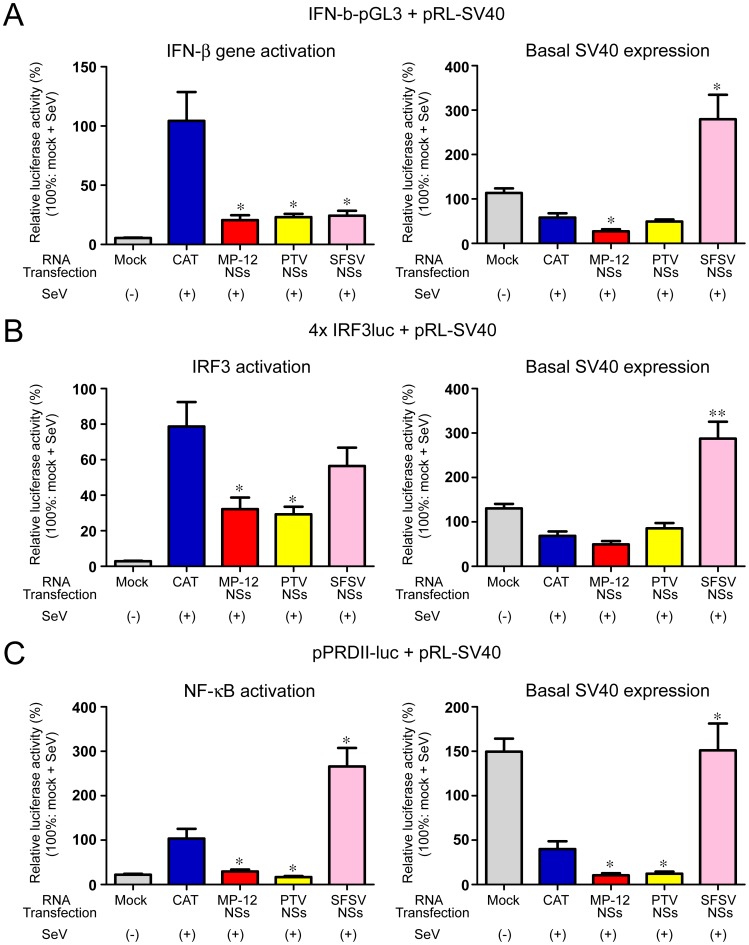
Inhibition of IFN-β promoter by PTV or SFSV NSs. 293 cells in 12-well plate were transfected with IFNb-pGL3 (A), 4×IRF3-luc (B) or pPRDII-luc plasmids (C) in addition to pRL-SV40 plasmid (transfection control). At 24 hours post transfection, cells were mock-infected or infected with SeV (30 U/well), and mock-transfected or immediately transfected with in vitro synthesized RNA encoding CAT (control) or NSs of MP-12, PTV Adames strain or SFSV. Cells were collected at 16 hpi, and the relative luciferase activity was measured by Dual-Luciferase Reporter Assay System (Promega, Madison, WI). Means+standard deviations of three independent experiments are shown in the graph. Asterisk represents statistical significance (Unpaired t-test, *p<0.05, **p<0.01, vs. CAT).

To further study these observations, we used dual luciferase reporter assays to analyze the activation of IFN-β promoter and two critical transcription factors for IFN-β gene upregulation; IFN regulatory factor-3 (IRF-3) and Nuclear Factor-Kappa B (NF-κB), in the presence of MP-12, PTV or SFSV NSs [63], [64]. 293 cells were transfected with (1) IFNb-pGL3 plasmids encoding firefly luciferase (FFluc) under the human IFN-β promoter [54], (2) 4×IRF3-luc plasmid encoding FFluc under 4 copies of the IFN-β promoter positive regulatory domain (PRD)I/III motif (IRF-3 binding element) [55], or (3) pPRDII-luc plasmids encoding FFluc under the IFN-β promoter PRDII motif (NF-κB-binding element) [56]. As a transfection control, pRL-SV40 plasmid encoding *Renilla* luciferase (rLuc) under the constitutively-active SV40 promoter was co-transfected with above plasmids. At 24 hours post transfection, cells were mock-infected or infected with 100 U/ml of Sendai virus (SeV), and immediately mock-transfected or transfected with *in vitro* synthesized RNA encoding either chloramphenicol acetyl transferase (CAT) (control) or NSs of MP-12, PTV Adames strain or SFSV. Cells were collected at 16 hpi, and the relative luciferase activity was measured. When we defined the FFluc activities obtained from SeV-infected cells transfected with IFNb-pGL3, 4×IRF3-luc or pPRDII-luc as 100%, FFluc activities of mock-infected cells transfected with IFNb-pGL3, 4×IRF3-luc or pPRDII-luc showed 5.5%, 2.8% or 22% of the SeV-infected cells, respectively (**Fig. 6 A-C** **, left panels, gray bars**). On the other hand, rLuc activities of mock-infected cells transfected with pRL-SV40 (control plasmid) were similar or increased compared to those of SeV-infected cells transfected with pRL-SV40 (**Fig. 6 A-C** **, right panels, gray bars**). The results suggest that SeV infection specifically induces the activation of IFN-β gene, IRF-3 and NF-κB. Compared to CAT RNA control (**Fig. 6 A-C** **, left panels, blue bars**), cells transfected with MP-12 NSs RNA reduced the FFluc expression level derived from the IFN-β promoter (**Fig. 6 A** **, left panels, red bars**), IRF-3 (**Fig. 6 B** **, left panels, red bars**) and NF-κB (**Fig. 6 C** **, left panels, red bars**). Our results were consistent with the findings that RVFV NSs inhibits the general transcription factor TFIIH and induces general transcription suppression regardless of IRF-3 or NF-κB activation [28], [29]. Similarly, cells transfected with NSs RNA of PTV Adames reduced the FFluc expression level derived from the IFN-β promoter (**Fig. 6 A** **, left panels, yellow bars**), IRF-3 (**Fig. 6 B** **, left panels, yellow bars**) and NF-κB (**Fig. 6 C** **, left panels, yellow bars**). Interestingly, cells transfected with SFSV NSs RNA consistently increased the rLuc activity derived from the constitutively-active SV40 promoter (**Fig. 6 A–C** **, right panels, pink bars**). SFSV NSs reduced FFluc expression level derived from the IFN-β promoter (**Fig. 6 A** **, left panels, pink bars**) and IRF-3 (**Fig. 6 B** **, left panels, pink bars**), but not that from NF-κB (**Fig. 6 C** **, left panels, pink bars**). These results suggest that MP-12 NSs and PTV Adames NSs inhibit the reporter activities derived from the IFN-β promoter, IRF-3 and NF-κB, while SFSV NSs inhibits reporter activities derived from the IFN-β promoter and IRF-3 but not those from NF-κB. In addition, SFSV NSs seems to increase the expression of constitutively active genes through a currently unknown mechanism.

### Immunogenicity and efficacy of MP-12 encoding NSs of PTV or SFSV

Based on the experiments described above, we confirmed that rMP12-PTNSs induces host general transcription suppression, inhibits the up-regulation of IFN-β gene, and does not promote PKR degradation, while rMP12-SFSNSs inhibits the up-regulation of IFN-β gene, but does not induce host general transcription suppression or PKR degradation. Next, we tested the efficacy of MP-12, rMP12-PTNSs or rMP12-SFSNSs against wt RVFV challenge in outbred mice. We also tested the previously reported rMP12-NSsR173A, which encodes mutant MP-12 NSs R173A [52]. Like rMP12-PTNSs, rMP12-NSsR173A inhibits host general transcription, suppresses activation of IFN-β gene but does not promote the degradation of PKR [52]. Five-week-old outbred CD1 mice were mock-vaccinated with PBS (n = 10), or subcutaneously vaccinated with 1×10^5^ pfu of MP-12 (n = 20), rMP12-NSR173A (n = 10), rMP12-PTNSs (n = 9) or rMP12-SFSNSs (n = 10). We used outbred mice to evaluate the immunogenicity and efficacy of mice with different genetic background. Mice were monitored daily, and intraperitoneally challenged with 1×10^3^ pfu of RVFV ZH501 strain at 45 days post vaccination. We performed mouse IFN-α ELISA using mouse sera at 1 day post vaccination as described previously [45]. At 1 day post vaccination, mice vaccinated with MP-12 or rMP12-PTNSs did not increase the level of IFN-α in sera, while serum IFN-α was detected in some mice vaccinated with rMP12-NSsR173A (33%) or rMP12-SFSNSs (10%) (data not shown). Low levels of viremia (100 pfu/ml) were detected in some mice vaccinated with MP-12 (20% at 3 days post vaccination), rMP12-R173A (10% at 2 days post vaccination) or rMP12-PTNSs (10% at 3 days post vaccination) (data not shown). One mouse vaccinated with MP-12 that suffered viremia was dead at 13 days post vaccination, and 1 mouse vaccinated with rMP12-SFSNSs became moribund, and was euthanized at 14 days post vaccination. We analyzed the euthanized mouse vaccinated with rMP12-SFSNSs histopathologically, and found no lesions in liver and spleen, while the encephalitis characterized by mild perivascular cuffing with mononuclear cells and neuronal necrosis with infiltration of microglia were observed. Viral N antigens were diffusely detected in the parenchymal area, and neurons in hippocampus, cortex and medulla contained viral antigens (**Fig. S1**). None of mice vaccinated with rMP12-NSsR173A or rMP12-PTNSs died before wt RVFV challenge. The result suggests that rMP12-SFSNSs retains neuroinvasiveness and neurovirulence similar to those of parental MP-12.

**Figure 7:**
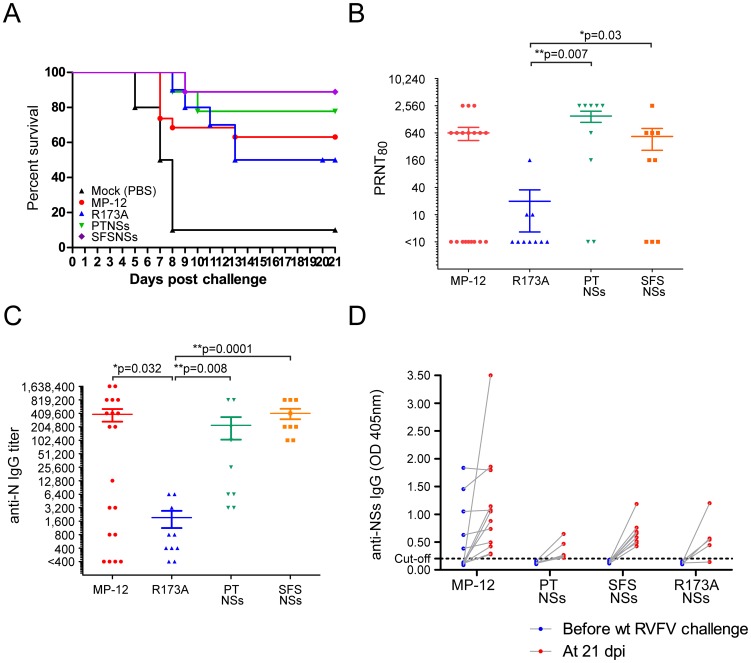
Efficacy and immunogenicity of rMP12-PTNSs or rMP12-SFSNSs in mice. Five-week-old CD1 mice were mock-vaccinated with PBS (n = 10) or vaccinated subcutaneously with 1×10^5^ pfu of MP-12 (n = 20), rMP12-NSsR173A (n = 10), rMP12-PTNSs (n = 9) or rMP12-SFSNSs (n = 10). Sera were collected at 42 days post vaccination, and mice were challenged with 1×10^3^ pfu of wt RVFV ZH501 strain (i.p) at 45 days post vaccination. Mice were observed for 21 days post-challenge. (A) Kaplan-Meier survival curves of vaccinated mice after wt RVFV challenge. (B) Neutralizing antibody titers of vaccinated mice (PRNT_80_). Asterisk represents statistical significance (Mann-Whitney U-test, *p<0.05, **p<0.01 vs. rMP12-NSsR173A). (C) Anti-N IgG titer measured by IgG ELISA. Y-axis shows endpoint titers of sera. Asterisk represents statistical significance (Mann-Whitney U-test, *p<0.05, **p<0.01 vs. rMP12-NSsR173A). (D) Anti-NSs IgG level measured by IgG ELISA. Y-axis shows OD405 nm of sera at 1∶100 dilutions, and cut-off at 0.204 is shown as dotted line.

In the challenge study, 90% of mock-vaccinated mice died within 8 days post infection, while 63%, 50%, 78% or 89% of mice vaccinated with MP-12, rMP12-NSR173A, rMP12-PTNSs or rMP12-SFSNSs were protected from wt RVFV challenge, respectively (**Fig. 7A**). The survival curve was statistically analyzed with Log rank test, and the difference in the survival curves among mice immunized with MP-12, rMP12-NSsR173A, rMP12-PTNSs or rMP12-SFSNSs were not statistically significant. A mock-vaccinated mouse that survived after wt RVFV challenge did not show reduction of body weight by more than 5% (**Fig. S2B**), and developed 1∶2,560 of neutralizing antibody at 21 days post wt RVFV challenge (data not shown), while 7 to 20% drop in body weight was observed before euthanasia in 80% of mock-vaccinated moribund mice after wt RVFV challenge (**Fig. S2A**).

Among the mice vaccinated with MP-12, 9 (47%) mice developed both neutralizing antibodies; plaque reduction neutralizing test (PRNT_80_) titer 1∶640 to 1∶2,560, and anti-N IgG titer 1∶800 to 1∶204,800 at 42 days post vaccination, and those mice all survived wt RVFV challenge (**Fig. 7B and C** **,** **Table 1**). The 9 surviving mice showed body weight at 90 to 110% range during the observation period (**Fig. S2C**). On the other hand, 10 (53%) mice did not develop neutralizing antibodies; 3 survived (**Fig. S2D**) and 7 died (**Fig. S2E and F**) after wt RVFV challenge. Among the 3 survivors, 2 developed anti-N IgG (1∶204,800) and the other (#14-3) did not raise anti-N IgG. The mouse #14-3 showed temporal 16% body weight reduction at 7 days post challenge (**Fig. S2D**), and also developed neutralizing antibodies (1∶2,560) at 21 days post challenge. On the other hand, rMP12-NSsR173A showed poor efficacy (50% survival) (**Table 1**). Three surviving mice (30%) vaccinated with rMP12-NSsR173A developed both neutralizing antibody (1∶10 to 1∶160) and anti-N IgG (1∶400 to 1∶6,400) (**Fig. S2G**), while 2 surviving mice (20%) had developed anti-N IgG (1∶800 to 1∶6,400) without neutralizing antibody (**Fig. S2H**). On the other hand, the remaining 5 mice (50%) died without the presence of neutralizing antibodies (3 mice developed 1∶400 to 1∶3,200 of anti-N IgG) (**Fig. S2I and J**). Together with previous observation that rMP12-NSsR173A does not efficiently accumulate viral proteins due to PKR-mediated eIF2α phosphorylation [52], this result suggests that MP-12 encoding a mutant NSs, which inhibits host general transcription including IFN-β gene, but does not promote PKR degradation, is not immunogenic, and poorly induces neutralizing antibodies.

**Table 1:** Antibody responses of mice vaccinated with MP-12 or the variants.

			42 days post vaccination		a-NSs IgG (OD_405_)	
Virus	Mouse #	Protection	PRNT_80_	a-N IgG	Pre	Post
MP-12	14-1	Dead (7 dpi)	-	3,200	0.11	-
	14-5	Dead (8 dpi)	-	3,200	0.11	-
	15-3	Dead (7 dpi)	-	409,600	0.10	-
	3-1	Dead (7 dpi)	-	800	0.14	-
	3-3	Dead (7 dpi)	-	-	0.12	-
	3-5	Dead (13 dpi)	-	-	0.14	-
	4-1	Dead (7 dpi)	-	-	0.12	-
	14-4	Survived	-	819,200	0.63*	0.74*
	15-2	Survived	-	1,638,400	0.12	0.29*
	14-3	Survived	-	-	0.11	3.50*
	14-2	Survived	2,560	12,800	0.13	1.15*
	15-4	Survived	2,560	800	0.12	0.28*
	15-5	Survived	2,560	1,638,400	0.38*	0.50*
	3-2	Survived	640	409,600	0.12	0.42*
	3-4	Survived	640	819,200	1.84*	1.74*
	4-2	Survived	640	409,600	0.13	0.88*
	4-3	Survived	640	204,800	0.09	1.05*
	4-4	Survived	640	204,800	1.05*	1.08*
	4-5	Survived	640	819,200	1.45*	1.86*
rMP12-PTNSs	16-2	Survived	2,560	6,400	0.13	0.25*
	16-3	Survived	2,560	6,400	0.12	0.23*
	16-4	Survived	160	25,600	0.11	0.27*
	17-1	Survived	2,560	819,200	0.12	0.65*
	17-2	Survived	640	102,400	0.11	0.22*
	17-3	Survived	2,560	819,200	0.10	0.47*
	17-4	Survived	2,560	204,800	0.12	0.25*
	17-5	Dead (8 dpi)	-	3,200	0.11	-
	16-1	Dead (10 dpi)	-	3,200	0.13	-
rMP12-SFSNSs	20-1	Survived	160	204,800	0.18	0.48*
	20-2	Survived	640	819,200	0.14	0.66*
	20-3	Survived	160	204,800	0.13	0.69*
	20-4	Survived	640	409,600	0.13	0.58*
	21-3	Survived	2,560	819,200	0.14	0.51*
	21-4	Survived	640	819,200	0.11	0.42*
	21-5	Survived	-	204,800	0.15	1.19*
	21-1	Survived	-	102,400	0.15	0.76*
	21-2	Dead (9 dpi)	-	102,400	0.14	-
rMP12-NSsR173A	7-1	Survived	160	400	0.10	0.57*
	7-4	Survived	10	800	0.13	0.14
	8-1	Survived	10	6,400	0.15	0.54*
	7-3	Survived	-	6,400	0.12	0.44*
	8-5	Survived	-	800	0.13	1.20*
	7-2	Dead (13 dpi)	-	400	0.12	-
	7-5	Dead (13 dpi)	-	400	0.11	-
	8-2	Dead (9 dpi)	-	3,200	0.11	-
	8-3	Dead (8 dpi)	-	-	0.10	-
	8-4	Dead (11 dpi)	-	-	0.10	-
PBS	12-4	Survived	-	-	0.13	1.92*

* a-NSs IgG positive (cut-off = 0.204).

Pre: 42 days post vaccination, Post: 21 days post wt RVFV challenge.

Compared to vaccination with MP-12 or rMP12-NSsR173A, all mice vaccinated with rMP12-PTNSs or rMP12-SFSNSs developed anti-N IgG (**Fig. 7C** **,** **Table 1**). Furthermore, mice vaccinated with rMP12-PTNSs or rMP12-SFSNSs showed significantly higher titers of neutralizing antibodies and anti-N IgG than those vaccinated with rMP12-NSsR173A (**Fig. 7B and C**). None of the survived mice vaccinated with rMP12-PTNSs or rMP12-SFSNSs showed a decrease in body weight below 90% (**Fig. S3**). The results suggest that rMP12-PTNSs and rMP12-SFSNSs have slightly higher efficacy than MP-12 and induce neutralizing antibodies at equivalent level to those induced by MP-12 in spite of a lack of PKR degradation function.

DIVA is important for vaccination of ruminants. Inclusion of negative DIVA marker helps to detect animals exposed to wt RVFV during outbreak. Unfortunately, MP-12 vaccine does not have a DIVA marker, and further improvement of MP-12 is required for an efficient detection of naturally infected animals during RVF outbreak. We determined whether mice vaccinated with rMP12-PTNSs or rMP12-SFSNSs can induce IgG reactive to RVFV NSs or not. For the purpose, we used IgG ELISA using C-terminus RVFV NSs [45]. We used the C-terminus NSs because the antigen is soluble, and detect anti-NSs antibody at higher sensitivity than IgG ELISA using purified whole RVFV NSs (**Fig. S4**). As shown in **Fig. 7D**, none of the mice vaccinated with rMP12-PTNSs or rMP12-SFSNSs induced detectable levels of IgG cross-reactive to the C-terminus of RVFV NSs, while 26% of mice vaccinated with MP-12 had detectable anti-NSs IgG in our IgG ELISA. The presence of anti-RVFV NSs antibody in vaccinated animals will compromise the DIVA strategy to detect animals exposed to wt RVFV. On the other hand, the rMP12-PTNSs and rMP12-SFSNSs did not induce anti-RVFV NSs antibody detectable in this IgG ELISA, and are applicable to DIVA. We also noted that all mice vaccinated with MP-12, rMP12-PTNSs or rMP12-SFSNSs raised anti-NSs IgG after wt RVFV challenge, suggesting the vaccinations do not confer sterile immunity, and wt RVFV replicated in vaccinated mice.

## Discussion

Live-attenuated Smithburn vaccine, generated by serial passage of wt RVFV Entebbe strain in mouse brain, has been used in endemic areas as a veterinary vaccine since 1950s, and its genetic subpopulations have never been reported. Grobbelaar et al. suggested that the use of Smithburn strain in endemic countries could result in the spread of RVFV strain by multiple use of automatic syringes for both viremic and uninfected ruminants during outbreaks, and may have resulted in reassortment of Smithburn strain with circulating wt RVFV during an outbreak [11]. Thus, a live-attenuated RVF vaccine that contains virulent subpopulations or non-attenuated segments, should not be used in viremic animals.

MP-12 vaccine strain was generated by serial 12-time plaque passages in MRC-5 cells in the presence of 5-fluorouracil [35]. The safety and immunogenicity of MP-12 in ruminants were reported [38], [39], [40], [41], and MP-12 is excluded from HHS/USDA select agent rule in the U.S. The MP-12 vaccine generated from the master seed currently has the Investigational New Drug (IND) status in the U.S., and was manufactured by using certified MRC-5 cells for human clinical trials [36]. We recently characterized the genetic subpopulations of an MP-12 vaccine lot, and found that the major population of MP-12 is highly stable and no reversions to the parental ZH548 strain were detected [36]. Different from viral passages in MRC-5 cells, RVFV induces NSs gene truncation during passages in cells lacking an intact type-I IFN system [43], [44]. The genetic stability and consistency of immunogenicity profiles are important factors in vaccine development, and the use of MRC-5 cells may be an important factor to maintain the original populations of MP-12 during manufacturing. One study also suggested that MP-12 strain with unknown passage history caused abortion and teratogenic effect in lambs when it was used for pregnant ewes at 35 to 56 days of pregnancy [65]. In this study, we aimed to develop MP-12 encoding functional NSs gene derived from serologically distinct phleboviruses to encode a DIVA marker in MP-12 while retaining the original efficacy and ability to efficiently replicate in MRC-5 cells. To encode a DIVA marker without affecting the ability to replicate in type-I IFN-competent MRC-5 cells, we designed MP-12 encoding serologically distinct phlebovirus NSs (PTV NSs or SFSV NSs), which is known to inhibit host IFN-β [34], [48]. The rMP12-PTNSs and rMP12-SFSNSs efficiently inhibited host IFN-β gene up-regulation induced by the MP-12 replication. Interestingly, rMP12-PTNSs but not rMP12-SFSNSs replicated efficiently in MRC-5 cells, indicating that rMP12-PTNSs can be considered as an alternative candidate vaccine of MP-12 with DIVA marker, which can be produced by using MRC-5 cells. Any MP-12 variants including rMP12-SFSNSs, which does not replicate in MRC-5 cells, will require genetic stability test in type-I IFN-incompetent cells to optimize the vaccine production.

PTV Adames strain [66] inhibits the human IFN-β gene [48]. SFSV NSs also inhibits the IFN-β gene expression if it is expressed from wt RVFV in place of RVFV NSs, while it does not promote the degradation of PKR [34], [47]. Consistent with wt RVFV encoding SFSV NSs, rMP12-SFSNSs inhibited the up-regulation of IFN-β gene and did not promote PKR degradation. We found that PTV NSs also does not promote PKR degradation (**Fig. 1**). Furthermore, PTV NSs inhibited host general transcription and the IFN-β promoter, while SFSV NSs inhibited the IFN-β promoter but not host general transcription (**Fig. 4** **–** **6**). Interestingly, rMP12-PTNSs formed clear plaques indistinguishable to those of parental MP-12, while rMP12-SFSNSs formed turbid plaques similar to those of rMP12-C13type (**Fig. 1B**) [51], [58].

RVFV NSs promotes the degradation of PKR [32], [34], and we previously found that the expression of dominant-negative PKR in place of MP-12 NSs increases the accumulation of dendritic cells infected with the MP-12 mutant [45]. On the other hand, the immunogenicity and efficacy of MP-12 encoding NSs mutant, which inhibits host general transcription but not PKR, have not been studied. The rMP12-NSsR173A inhibits host general transcription including IFN-β gene without promoting the degradation of PKR [52]. In this study, we found that rMP12-PTNSs inhibits host general transcription including IFN-β gene, and does not promote the degradation of PKR. Mice vaccinated with rMP12-PTNSs but not rMP12-NSsR173A induced high level of neutralizing antibodies. The result suggested that the host transcription suppression induced by RVFV NSs negatively affects the vaccine efficacy if PKR is not inhibited. On the other hand, MP-12 encoding PTV NSs was highly efficacious even though it induces host general transcription suppression without inducing PKR degradation. It might be possible that PTV NSs has other unknown functions to support RVFV replication in the presence of host general transcription suppression. We noted that SFSV NSs possesses an unknown function to increase host gene expression, as indicated by up-regulation of a constitutively expressed SV40 reporter gene (**Fig. 6**). The gene up-regulation was induced independently of PKR degradation, and may contribute to consistently high level of anti-N IgG in rMP12-SFSNSs vaccinated mice. Further studies are currently being conducted in our laboratory to elucidate the detailed mechanism of PTV NSs and SFSV NSs in host gene expression. Both SFSV and PTV cause self-limiting febrile illness in humans, and no significant diseases in animals. We observed that one mouse vaccinated with rMP12-SFSNSs was dead at 9 days post vaccination with viral encephalitis (Fig. S1). Vaccine-related viral encephalitis was also observed in mice vaccinated with parental MP-12 (data not shown), and we did not observe any significant increase of mouse death related to rMP12-SFSNSs vaccination compared to MP-12 vaccination. Considering that MP-12 vaccine encodes fully functional NSs of RVFV, rMP12-PTNSs and rMP12-SFSNSs are similar with, or most probably more attenuated than parental MP-12 due to a lack of function to promote PKR degradation. Since mouse is the most susceptible species to RVFV infection, the vaccine safety should be test in ruminants and nonhuman primates before further consideration as a vaccine candidate.

In summary, MP-12 mutants encoding NSs of PTV or SFSV are highly efficacious in mice and encode a DIVA marker, while rMP12-PTNSs also replicates efficiently in MRC-5 cells, which is useful for the vaccine manufacturing process. The immunogenicity and safety profile of rMP12-PTVNSs in ruminants and nonhuman primates will need to be tested to develop this virus as an alternative of MP-12 vaccine for veterinary and human use, respectively.

## Supporting Information

Figure S1 **Viral antigen distributions in dead mouse vaccinated with rMP12-SFSNSs.** Immunohistochemistry with anti-RVFV N antibody in (A) Hippocampus CA2 region, (B) Occipital cortex, (C) Medulla, (D) Liver and (E) Spleen. Cells reacting to anti-RVFV N antibody are indicated by arrows. Tissues of the euthanized mouse were fixed with 10% buffered formalin, and paraffin blocks were generated for pathological evaluation. Sections were incubated for 2 hours with anti-RVFV N rabbit polyclonal antibody [45], followed by incubating 30 min with biotinylated anti rabbit IgG (BA-1000, Vector Laboratory, CA). Signals were detected by the labeled streptavidin-biotin method with a UltraVision Alk-Phos kit (TS-060-AP, Thermo Scientific, CA). Vector Red Alkaline Phosphatase substrate (SK-5100, Vector Laboratory, CA) was used as chromogen, and counter-staining was performed with hematoxylin. Reagent negative controls consisted of samples in which primary antibody was replaced with rabbit IgG were included for confirming the specificity of reaction (data not shown).
(PDF)

Figure S2 **Body weight changes of mice mock-vaccinated or vaccinated with MP-12 or rMP12-NSsR173A.** Body weight of mice was monitored from 0 to 21 days post wt RVFV ZH501 challenge. (A) mock-vaccinated dead mice, (B) mock-vaccinated survived mouse, (C) MP-12 vaccinated survived mice with neutralizing antibodies, (D) MP-12 vaccinated survived mice without neutralizing antibodies, (E) MP-12 vaccinated dead mice; neutralizing antibodies (−) and anti-N IgG (+), (F) MP-12 vaccinated dead mice; neutralizing antibodies (−) and anti-N IgG (−), (G) rMP12-R173A vaccinated survived mice with neutralizing antibodies, (H) rMP12-R173A vaccinated survived mice without neutralizing antibodies, (I) rMP12-R173A vaccinated dead mice; neutralizing antibodies (−) and anti-N IgG (+), (J) rMP12-R173A vaccinated dead mice; neutralizing antibodies (−) and anti-N IgG (−). Body weight was normalized (100%) to that at 0 day post wt RVFV challenge.
(PDF)

Figure S3 **Body weight changes of mice vaccinated with rMP12-PTNSs or rMP12-SFSNSs.** Body weight of mice was monitored from 0 to 21 days post wt RVFV ZH501 challenge. (A) rMP12-PTNSs vaccinated survived mice with neutralizing antibodies, (B) rMP12-PTNSs vaccinated dead mice without neutralizing antibodies, (C) rMP12-SFSNSs vaccinated survived mice with neutralizing antibodies, (D) rMP12-SFSNSs vaccinated survived or dead mice without neutralizing antibodies. Body weight was normalized (100%) to that at 0 day post wt RVFV challenge.
(PDF)

Figure S4 **Sensitivity of IgG ELISA using GST-NSs (C-terminus).** The sensitivity of IgG ELISA using purified GST-RVFV NSs (C-terminus) or purified Nus-RVFV NSs (full-length NSs with N-terminal Nus-tag expressed in E.coli by using pET-43.1b [Novagen]) to detect anti-NSs IgG by using (A) a mouse serum raised against mouse brain infected with RVFV (kind gift from Dr. Tesh, UTMB) or (B) anti-NSs antibody, which was raised against peptide encoding the C-terminus (EESDDDGFVEVD) of RVFV NSs in rabbit (EZ BioLab). Purified GST and Nus were used as control antigens. The 96-well plate was coated with GST, GST-NSs, Nus, Nus-NSs (100 ng per well), and the reactivity of each antibody (2-fold dilutions) was measured by ELISA as described in materials and method.
(PDF)
