# Supplementary figures and images for: Characterization of Rift Valley Fever Virus MP-12 Strain Encoding NSs of Punta Toro Virus or Sandfly Fever Sicilian Virus
Source: PLoS Negl Trop Dis. 2013 Apr 18;7(4):e2181. doi: 10.1371/journal.pntd.0002181 (PMC3630143; doi:10.1371/journal.pntd.0002181)

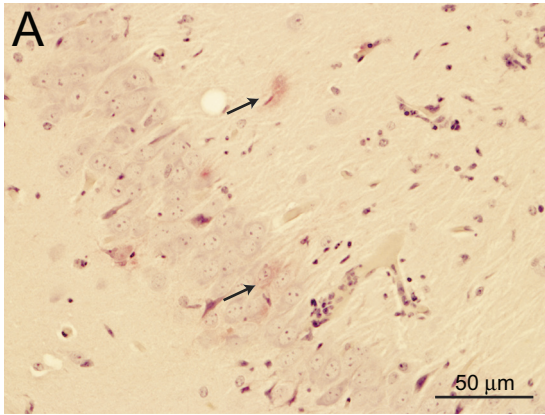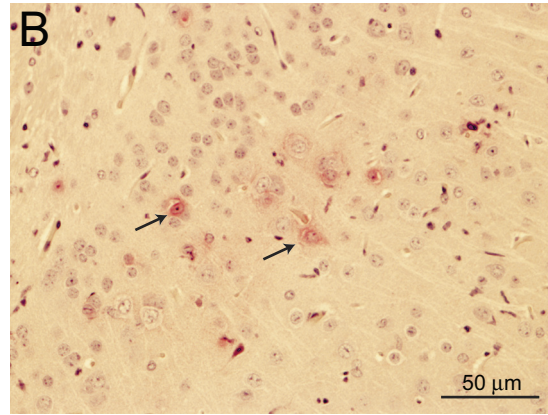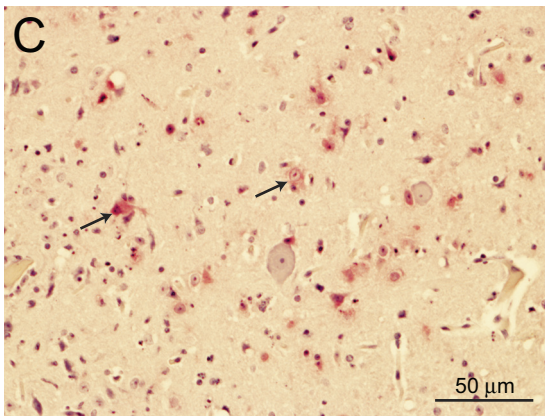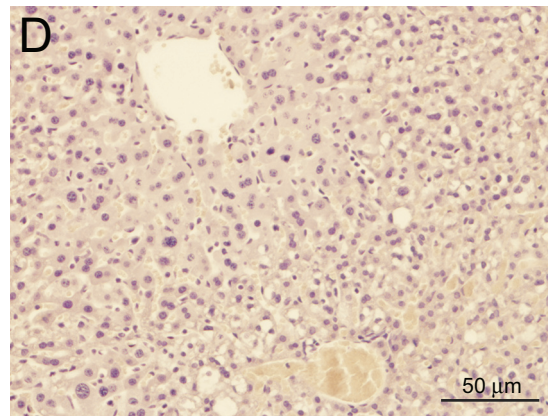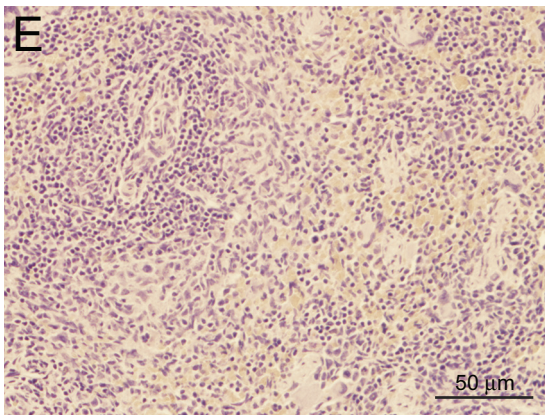

Supplement: Figure S1 — Viral antigen distributions in dead mouse vaccinated with rMP12-SFSNSs. Immunohistochemistry with anti-RVFV N antibody in (A) Hippocampus CA2 region, (B) Occipital cortex, (C) Medulla, (D) Liver and (E) Spleen. Cells reacting to anti-RVFV N antibody are indicated by arrows. Tissues of the euthanized mouse were fixed with 10% buffered formalin, and paraffin blocks were generated for pathological evaluation. Sections were incubated for 2 hours with anti-RVFV N rabbit polyclonal antibody [45], followed by incubating 30 min with biotinylated anti rabbit IgG (BA-1000, Vector Laboratory, CA). Signals were detected by the labeled streptavidin-biotin method with a UltraVision Alk-Phos kit (TS-060-AP, Thermo Scientific, CA). Vector Red Alkaline Phosphatase substrate (SK-5100, Vector Laboratory, CA) was used as chromogen, and counter-staining was performed with hematoxylin. Reagent negative controls consisted of samples in which primary antibody was replaced with rabbit IgG were included for confirming the specificity of reaction (data not shown). (PDF) [file pntd.0002181.s001.pdf]

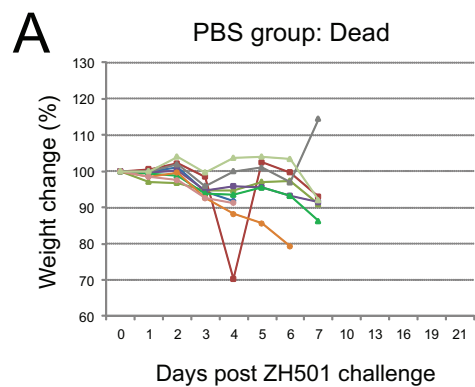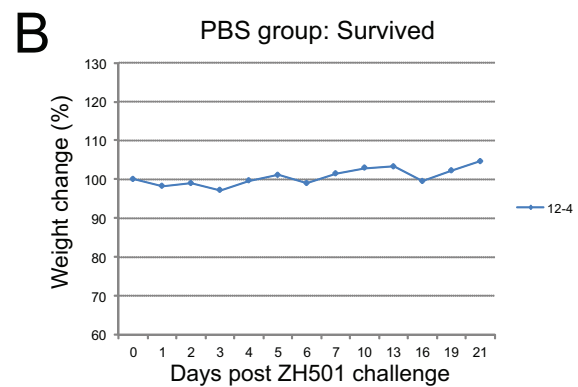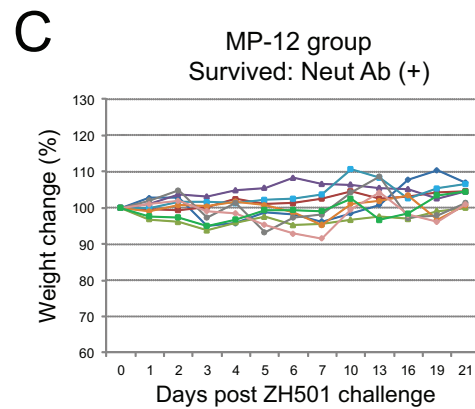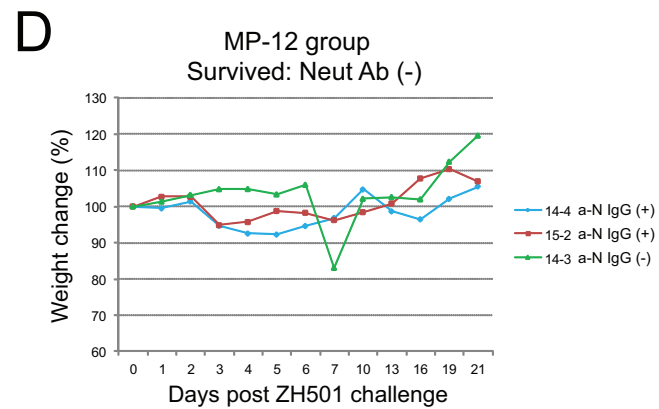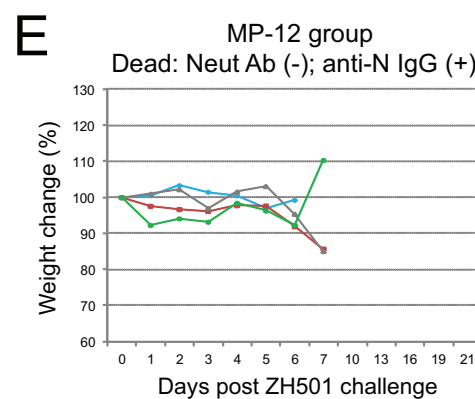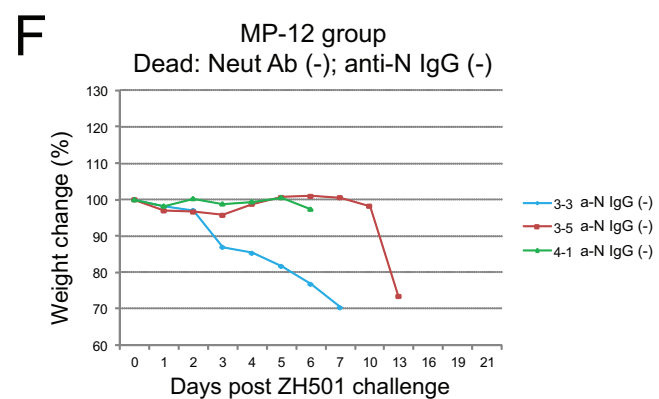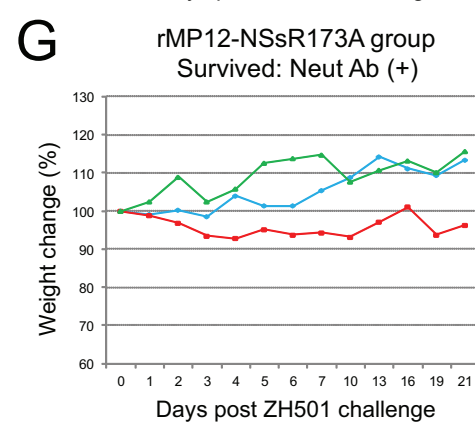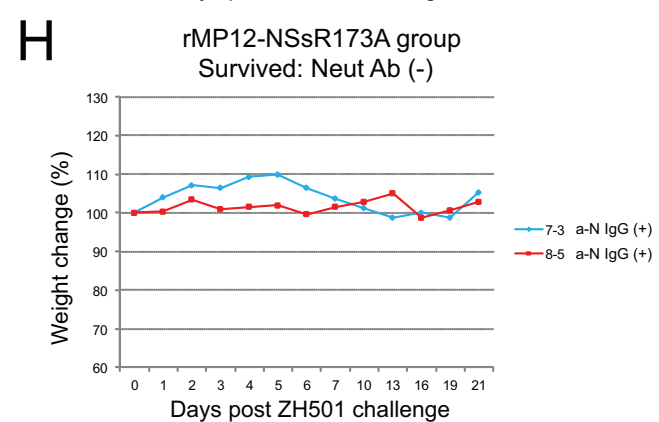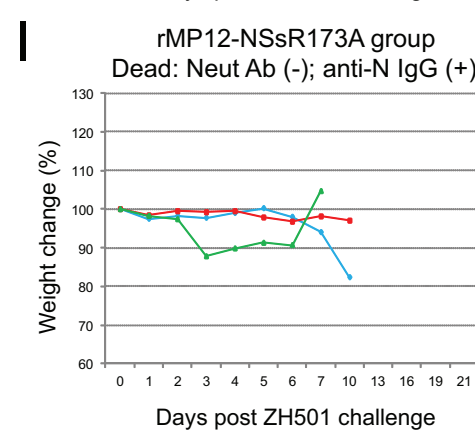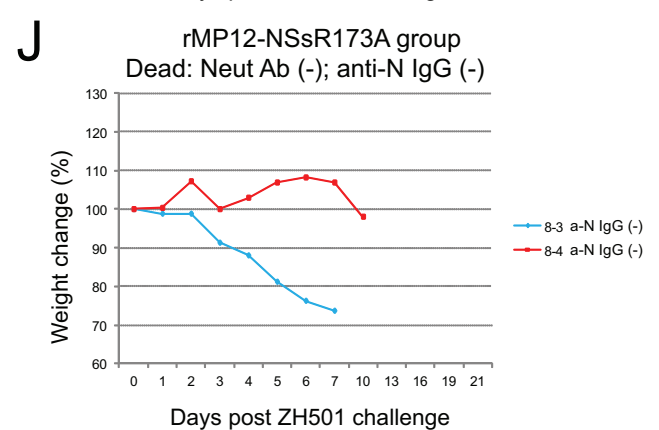

Supplement: Figure S2 — Body weight changes of mice mock-vaccinated or vaccinated with MP-12 or rMP12-NSsR173A. Body weight of mice was monitored from 0 to 21 days post wt RVFV ZH501 challenge. (A) mock-vaccinated dead mice, (B) mock-vaccinated survived mouse, (C) MP-12 vaccinated survived mice with neutralizing antibodies, (D) MP-12 vaccinated survived mice without neutralizing antibodies, (E) MP-12 vaccinated dead mice; neutralizing antibodies (−) and anti-N IgG (+), (F) MP-12 vaccinated dead mice; neutralizing antibodies (−) and anti-N IgG (−), (G) rMP12-R173A vaccinated survived mice with neutralizing antibodies, (H) rMP12-R173A vaccinated survived mice without neutralizing antibodies, (I) rMP12-R173A vaccinated dead mice; neutralizing antibodies (−) and anti-N IgG (+), (J) rMP12-R173A vaccinated dead mice; neutralizing antibodies (−) and anti-N IgG (−). Body weight was normalized (100%) to that at 0 day post wt RVFV challenge. (PDF) [file pntd.0002181.s002.pdf]

A

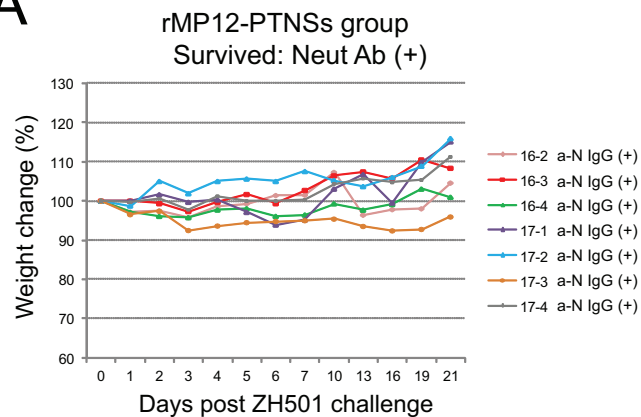

B

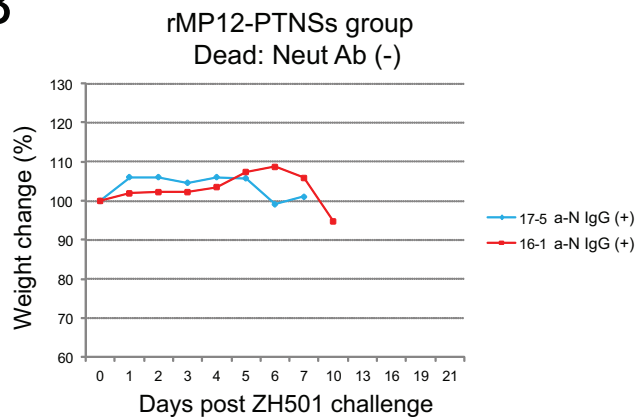

C

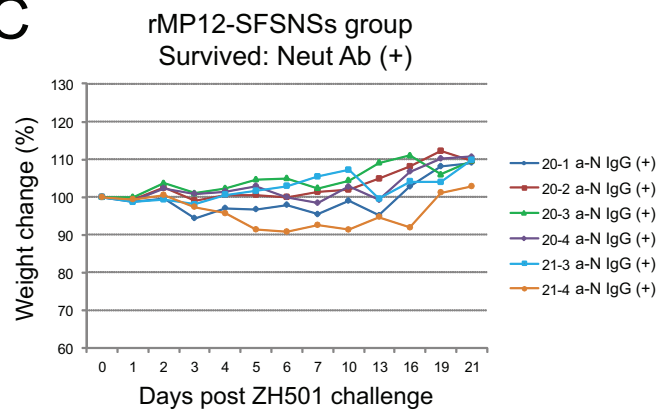

D

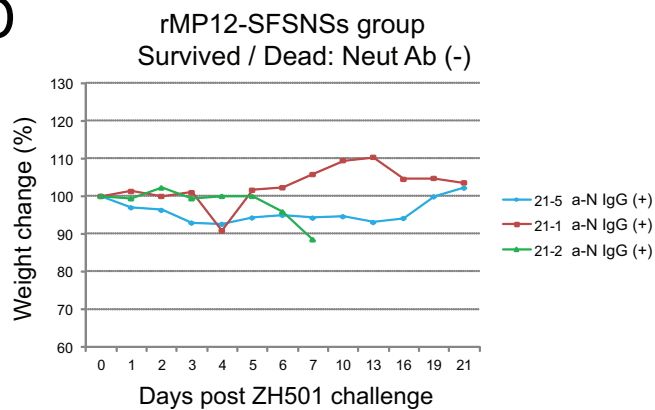

Supplement: Figure S3 — Body weight changes of mice vaccinated with rMP12-PTNSs or rMP12-SFSNSs. Body weight of mice was monitored from 0 to 21 days post wt RVFV ZH501 challenge. (A) rMP12-PTNSs vaccinated survived mice with neutralizing antibodies, (B) rMP12-PTNSs vaccinated dead mice without neutralizing antibodies, (C) rMP12-SFSNSs vaccinated survived mice with neutralizing antibodies, (D) rMP12-SFSNSs vaccinated survived or dead mice without neutralizing antibodies. Body weight was normalized (100%) to that at 0 day post wt RVFV challenge. (PDF) [file pntd.0002181.s003.pdf]

**A**

anti-RVFPV Ab

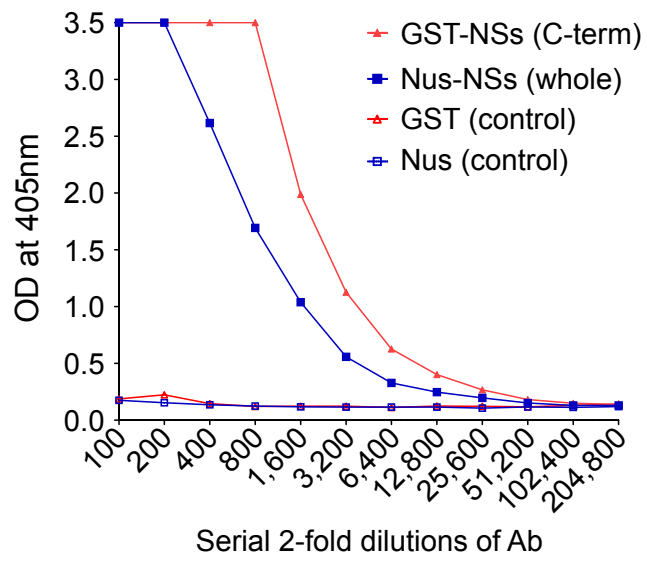**B**

anti-NSs Ab

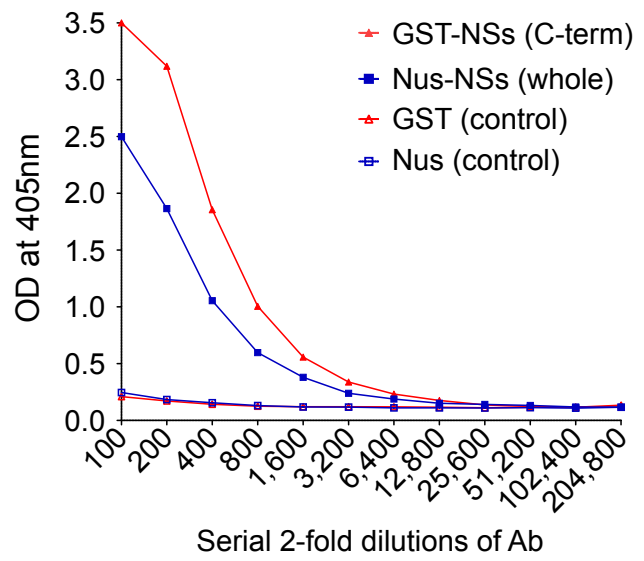

Supplement: Figure S4 — Sensitivity of IgG ELISA using GST-NSs (C-terminus). The sensitivity of IgG ELISA using purified GST-RVFV NSs (C-terminus) or purified Nus-RVFV NSs (full-length NSs with N-terminal Nus-tag expressed in E.coli by using pET-43.1b [Novagen]) to detect anti-NSs IgG by using (A) a mouse serum raised against mouse brain infected with RVFV (kind gift from Dr. Tesh, UTMB) or (B) anti-NSs antibody, which was raised against peptide encoding the C-terminus (EESDDDGFVEVD) of RVFV NSs in rabbit (EZ BioLab). Purified GST and Nus were used as control antigens. The 96-well plate was coated with GST, GST-NSs, Nus, Nus-NSs (100 ng per well), and the reactivity of each antibody (2-fold dilutions) was measured by ELISA as described in materials and method. (PDF) [file pntd.0002181.s004.pdf]
